# A Facebook-Delivered Weight Loss Intervention Using Open Enrollment: Randomized Pilot Feasibility Trial

**DOI:** 10.2196/33663

**Published:** 2022-05-06

**Authors:** Sherry L Pagoto, Matthew W Schroeder, Ran Xu, Molly E Waring, Laurie Groshon, Jared M Goetz, Christie Idiong, Haley Troy, Joseph DiVito, Richard Bannor

**Affiliations:** 1 Department of Allied Health Sciences Institute for Collaboration in Health, Interventions, and Policy University of Connecticut Storrs, CT United States

**Keywords:** weight loss, obesity, social media, Facebook, social networking, mobile phone

## Abstract

**Background:**

Behavioral weight loss programs typically enroll 12-40 people into groups that then suffer from declining engagement over time. Web-based patient communities, on the other hand, typically offer no limits on capacity and membership is fluid. This model may be useful for boosting engagement in behavioral weight loss interventions, which could lead to better outcomes.

**Objective:**

In this study, we aimed to examine the feasibility and acceptability of continuously enrolling participants into a Facebook-delivered weight loss intervention for the first 8 of 16 weeks relative to the same intervention where no new participants were enrolled after randomization.

**Methods:**

We conducted a randomized pilot trial to compare a Facebook weight loss group that used open enrollment with a group that used closed enrollment on feasibility and acceptability in adults with BMI 27-45 kg/m^2^. The feasibility outcomes included retention, engagement, and diet tracking adherence. We described the percentage loss of ≥5% weight in both groups as an exploratory outcome. We also explored the relationship between total volume of activity in the group and weight loss. The participants provided feedback via web-based surveys and focus groups.

**Results:**

Randomized participants (68/80, 85% women) were on average, aged 40.2 (SD 11.2) years with a mean BMI of 34.4 (SD 4.98) kg/m^2^. We enrolled an additional 54 participants (50/54, 93% female) in the open enrollment condition between weeks 1 and 8, resulting in a total group size of 94. Retention was 88% and 98% under the open and closed conditions, respectively. Randomized participants across conditions did not differ in engagement (*P*=.72), or diet tracking adherence (*P*=.42). Participant feedback in both conditions revealed that sense of community was what they liked most about the program and not enough individualized feedback was what they liked the least. Weight loss of ≥5% was achieved by 30% (12/40) of the participants randomized to the open enrollment condition and 18% (7/40) of the participants in the closed enrollment condition. Exploratory analyses revealed that the open condition (median 385, IQR 228-536.5) had a greater volume of engagement than the closed condition (median 215, IQR 145.5-292; *P*=.007). Furthermore, an increase of 100 in the total volume of engagement in the Facebook group each week was associated with an additional 0.1% weekly weight loss among the randomized participants (*P*=.02), which was independent of time, individual participant engagement, and sociodemographic characteristics.

**Conclusions:**

Open enrollment was as feasible and acceptable as closed enrollment. A greater volume of engagement in the Facebook group was associated with weight loss, suggesting that larger groups that produce more engagement overall may be beneficial. Future research should examine the efficacy of the open enrollment approach for weight loss in a fully powered randomized trial.

**Trial Registration:**

ClinicalTrials.gov NCT02656680; https://clinicaltrials.gov/ct2/show/NCT02656680

## Introduction

### Background

Obesity, a serious risk factor for cardiovascular disease and type 2 diabetes, affects 42.4% of the adults in the United States [1]. Robust evidence supports the efficacy of lifestyle interventions [2], but such interventions require regular in-person visits for 6-12 months, which is inconvenient for many people and a difficult model to scale up. Technology-delivered lifestyle interventions have been developed to address these challenges. Although weight loss outcomes from technology-delivered lifestyle interventions are promising, they still need optimization because weight loss outcomes tend to be lower than those produced by traditional clinic-based interventions [3]. Technology-based interventions are typically delivered via a web-based platform or mobile app in which participants receive counseling, peer support, and multimedia intervention content. Some of these interventions use commercial social media platforms, such as Facebook, because they are free, many people already use them, and some allow users to create private groups [4,5].

Systematic reviews and meta-analyses have revealed promising outcomes of social media-delivered lifestyle interventions [6-10], with modest but significant weight loss [11]. Engagement, defined as any visible activity in the group (eg, posts, comments on posts, *likes*, and poll votes), appears to be an important predictor of outcomes [12-15]. The degree of engagement reported in studies of social media-delivered interventions is highly variable, ranging from an average of once per participant during the entire intervention to 11 times per week per participant during the intervention [12,13,15-21]. Regardless of how engaged the participants are at the beginning of these programs, engagement generally declines over time. For example, in a study of a social media–delivered weight loss intervention, engagement in the first 3 months was significantly higher than that in the last 3 months [18]. The same study found that every 10 posts made by a participant corresponded to a −0.5% weight loss. Similarly, findings from a social media–delivered smoking cessation intervention study revealed that a 1-unit increase in engagement was associated with a 0.56-unit decrease in cigarettes smoked per week [22]. These findings suggest that keeping participants engaged throughout the course of the intervention may improve outcomes. Effective engagement strategies are required to accomplish this goal.

Few studies have tested strategies for increasing engagement in social media–delivered interventions. In our previous study of a Facebook-delivered weight loss intervention, participants were randomized to a condition in which a small number of participants were incentivized to engage daily or a condition that involved no incentivized engagement [15]. Participants in the incentivized condition were unaware of this arrangement until they were debriefed at the end of the intervention. Engagement was higher in the incentivized condition, but this was driven mostly by an increase in *likes* as opposed to posts and comments, which means that the activity of incentivized engagers did not effectively prompt others to speak up more in the group. This study was not powered to detect group differences in weight loss; however, greater engagement was associated with greater weight loss.

An alternative approach to increasing engagement might be to design web-based lifestyle interventions that are more similar to spontaneously formed web-based patient communities that tend to be highly engaged [23,24]. Web-based patient communities have become increasingly popular on commercial social media platforms, but they have also been developed by commercial digital health companies (eg, WW [formerly Weight Watchers]), nonprofit organizations (eg, the American Diabetes Association), and health care systems (eg, Mayo Clinic Connect) [25]. Web-based patient communities have been created for a wide variety of medical conditions, including diabetes [26], cancer [23], and cardiovascular disease [25]. Many of these communities have thousands of members and a high volume of daily engagement [27]. Spontaneously formed web-based patient communities are different from web-based communities created for behavioral programs, as the latter tend to recruit smaller groups of people who start and finish the program together on specific dates [12,13,17,28]. Spontaneously formed web-based patient communities are also larger and fluid in membership, such that new members can join any time, which allows them to grow quite large over time. Such communities might stay highly engaged because even if some members inevitably disengage over time, new members are always joining and keeping the discussion threads populated, which provides new content to read and respond to all members. Fluidity in web-based communities can result in a greater exchange of information, support, and resources, leading to more innovative knowledge creation among community members [29]. Further, the volume of daily engagement often remains consistently high for long periods, unlike the usual steady decline in engagement observed in short-term (eg, 3-6 months) web-based communities created to implement behavioral programs.

A high volume of engagement seems important for improving the impact of behavioral interventions delivered in web-based communities for 3 reasons. First, Facebook’s newsfeed algorithm prioritizes groups that show higher engagement [30]. This means that Facebook users are more likely to see posts in their newsfeeds from a highly engaged group than from a group where only a few members are engaging. Second, a higher volume of posts may simply produce more opportunities for members to engage, receive information and support, and feel connected to each other. Third, highly engaged groups may make engagement easier for timid members who are not comfortable being the first to speak up. In our previous work, we found that in postintervention interviews, participants expressed that they wished more participants engaged and felt uncomfortable being the first to speak up [12]. That said, too much content from a large community may cause members to feel cognitive overload [31] or make it difficult to find program content. An important difference between organically grown web-based communities and the ones researchers create to deliver behavioral interventions is that the former do not have a feed of behavioral intervention posts by a professional counselor; instead, the content is largely member generated. If web-based communities that are created to deliver behavioral interventions become saturated with participant-generated content, intervention receipt, defined as the degree to which participants saw intervention posts, could be compromised, which could then negatively impact outcomes. Research is needed to determine how to create a web-based community to deliver a lifestyle intervention in which participants are highly engaged but not so much that they feel that other participants’ posts impede their ability to follow the program.

### Goal of This Study

The purpose of the present *proof of concept* pilot study is to test the feasibility and acceptability of a Facebook-delivered lifestyle intervention that shares the open enrollment feature of organically grown web-based patient communities. As such, once a core set of participants is randomized into a group, enrollment continues to allow new people to join each week. Participants with overweight or obesity were randomized to receive either a Facebook-delivered lifestyle intervention in which enrollment continued for the first 8 of 16 weeks (ie, open enrollment condition) or the same intervention but in a Facebook group that did not continuously enroll participants (ie, closed enrollment condition). Our first aim was to examine retention (ie, percentage of participants providing their weight at follow-up) and acceptability (ie, percentage of participants who would recommend the program to a friend, percentage of participants who did not feel other participants posted too much, and percentage of participants who felt the counselors were responsive) in each condition and overall. We hypothesized that retention and acceptability would exceed our benchmark of 80% in both conditions. We measured acceptability quantitatively and qualitatively in focus groups, where participants were asked what they liked and disliked about the program and their thoughts on the size of their group.

The second aim was to compare the conditions on participant engagement during the intervention. Engagement was conceptualized in 4 ways. The first way was mean total engagement (ie, reactions, comments, posts, and poll votes) per randomized participant during the intervention. The second way was total engagement among all the participants in each condition during the intervention. The third way was total engagement produced by everyone in the group (all participants and counselors). The fourth way was engagement among all participants during the 1 year following the intervention when we left the groups open for participants to use as they wished. We hypothesized that the open enrollment condition would outperform the closed enrollment condition in all 4 metrics of engagement.

Our third aim was to compare the conditions on the number of complete daily diet records, a key behavioral weight loss strategy, to explore whether an increasingly populated group either motivates or distracts participants from their diet tracking.

Our fourth aim was to compare the conditions on counselors’ total engagement. We hypothesized that counselors in the open enrollment condition would engage more than counselors in the closed enrollment condition. This difference will inform future randomized trials on the cost-effectiveness of these intervention approaches.

Our fifth aim was to describe the percentage of weight loss from baseline to 16 weeks and the proportion of participants who lost ≥5% of their baseline weight in both conditions. This aim is descriptive because this pilot feasibility study did not have the power to detect significant differences in weight loss between conditions. Our sixth aim was to explore whether the volume of engagement in the Facebook group (ie, posts and comments from participants and counselors) each week was associated with weight loss among randomized participants. This sheds light on whether greater engagement in the overall group is a potential predictor of better outcomes.

## Methods

### Study Design, Settings, and Participants

This study was a pilot randomized feasibility trial in which 80 participants who were either overweight or obese were randomized into 1 of 2 remotely delivered lifestyle interventions. We recruited adults interested in losing weight via advertisements on the web, at the University of Connecticut, ResearchMatch, and yard sale and neighborhood Facebook groups in 37 states across the United States between June and October 2019. Inclusion criteria included having a BMI between 27 and 45 kg/m², owning a smartphone, being an active Facebook user (ie, comments or posts more than once a week), aged 18-65 years, and having daily internet access. Exclusion criteria were pregnancy, bariatric surgery or plans during the study period, loss of >5% weight in the past 3 months, pre-existing conditions that precluded physical activity or dietary changes, taking medications affecting weight, inability to walk a quarter mile without stopping, type 1 or type 2 diabetes, Participation in previous weight loss studies led by the principal investigator, inability to attend the orientation webinar, inability to provide consent, and refusal to be audiotaped (focus group).

Participants completed an orientation webinar before randomization, the purpose of which was to educate participants about participating in research, review study procedures, discuss the importance of follow-up data regardless of individual outcomes, and discuss barriers to participation [32]. Upon completion of the webinar, those still interested in participating in the study were mailed a Wi-Fi scale (Fitbit Aria) and asked to provide the staff with log-in information for the scale to record the weights for the assessments. We randomized 80 participants to the 2 conditions and continued to recruit participants for 7 weeks, placing all new recruits into the open enrollment condition. Each week, new recruits were introduced to the group by a counselor in a welcome post on Sunday evening.

### Intervention Conditions

#### Overview

Participants were randomized to either a Facebook group in which new participants were continually enrolled during weeks 1-8 (open enrollment) or a Facebook group that included only the original 40 randomized participants (closed enrollment). In the open enrollment condition, 54 additional participants were enrolled between weeks 1 and 8, for a final group size of 94 (open enrollment additional). Both Facebook groups were led by a registered dietitian, whose role was to provide counseling and support during the program. The dietitians in each group had a junior coleader who assisted them. All counselors completed the web-based Diabetes Prevention Program (DPP) Lifestyle Intervention training.

#### Facebook-Delivered Lifestyle Intervention

All participants received an identical 16-week lifestyle intervention based on the DPP [33] but modified to be delivered to a private Facebook group. We adapted the DPP content to be appropriate for a web-based setting as described elsewhere [34]. Each participant received an individualized calorie goal that would facilitate a 1 to 2 lb (0.45-0.91 kg) weight loss weekly and was asked to use MyFitnessPal to track their calories daily. They were asked to have the counselor review 2 weeks of their MyFitnessPal records but could request reviews more often as desired. The Facebook group was private, such that only those invited by the study team could join, and the group and all its content were viewable only to the members. Consistent with our previous work [34,35], the lifestyle intervention was delivered through twice-daily posts, with each week’s content reflecting 1 DPP module. The DPP goals include (1) calorie tracking based on achieving the calorie goal that corresponds to losing 1-2 lbs a week (0.45-0.91 kg), (2) following a healthy diet consistent with the American Heart Association guidelines [36], (3) getting 150 to 300 minutes per week of moderate or higher intensity exercise, and (4) strength training goal of 2 times per week according to the National Guidelines for Physical Activity [37]. On Mondays in the group, participants were instructed to set 2-3 diet- and exercise-related goals for the week. On Friday mornings, they were asked to report the degree to which their weight had changed in the past week (eg, lost 1 lb), but not their actual weight (eg, 250 lbs). On Sundays, they were asked to report whether they accomplished their diet and exercise goals for the week and if they did not, to engage in problem solving of the barriers. The remaining posts each week were related to that week’s DPP module (eg, Get More Active and Cope with Triggers). Throughout the intervention, staff produced weight and engagement reports for counselors to track which participants had not engaged during that week or had not lost weight. This allowed counselors to re-engage participants by tagging them in posts or sending private messages to check on them.

#### Focus Groups

At the end of the intervention, all participants were contacted by email to schedule a focus group via videoconference. Participants were asked what they liked most and least about the program and their opinions on how to improve various aspects of the program. The focus groups were recorded and transcribed.

#### Postintervention Period

At the end of the 16-week intervention, participants in both groups were informed that they may stay in the group for up to a year to continue using the group as they wished to support each other’s continued weight loss efforts; however, the counselor would no longer be present. In the final weeks of the intervention, the counselor in each group asked a volunteer to take over the group moderator role for this period. Each group had a volunteer who was willing to take on this role. They were also reminded that the study team would extract engagement data in the subsequent year as part of the research procedure. We tracked engagement in the year following the end of the intervention to see if the entire open enrollment group (randomized and additional) continued to engage to a greater degree than did the closed enrollment group.

### Measures

#### Retention

Retention was assessed by recording the percentage of participants in each condition who completed the 16-week follow-up assessment, which included the final weigh-in and survey.

#### Acceptability

Participants rated the acceptability of their intervention in the follow-up survey using the following items. First, participants rated how likely they would be to recommend the program to a friend using responses on a 5-point Likert scale from not at all likely to very likely. Second, participants reported what they thought of the amount of posts by other participants in the group using the following response options, “I would prefer that participants did not post at all,” “I would prefer fewer posts by participants,” “I liked the amount of posts by participants,” and “I would prefer more posts by participants.” Third, participants reported what they thought of the amount of comments made by other participants in their group using the following response options: “I would prefer that participants did not reply/comment at all,” “I would prefer fewer comments by participants,” “I liked the amount of comments by participants,” and “I would prefer more comments by participants.” Fourth, participants rated how responsive counselors were to participants’ posts using the following response options, “The counselors were not responsive,” “The counselors were somewhat responsive,” and “The counselors were very responsive.” Finally, we asked participants if they had become Facebook friends with any participants (yes or no), and if so, how many.

#### Engagement

Engagement was broadly defined as posts, comments, reactions (eg, love, wow, like, angry, and sad), and votes on polls. After the intervention was complete, we extracted the engagement data of the private Facebook groups using the Grytics app, except for poll data, which were extracted manually because Grytics does not extract poll data. We summarized the total number of posts, comments, reactions, and poll votes per randomized participant, as well as per participant (randomized and postrandomized) during the intervention period. We also calculated total participant engagement in each condition during the year following the intervention. In addition, we calculated the total volume of participant engagement as the total number of posts, comments, and poll votes in each condition (and in each week), and then the total volume of engagement, including that generated by the counselor and all participants in the group. We also summarized the posts, comments, reactions, and total engagement by counselors in each condition.

#### Diet Tracking Adherence

Diet tracking adherence was defined as the number of days a participant tracked their dietary intake in the MyFitnessPal app. A complete day of diet tracking was defined as any day on which the participants tracked ≥2 meals and ≥800 kcal per day [21,22].

#### Weight

Weight was collected at baseline and each week of the intervention via the Fitbit Aria scales the participants received upon enrollment. Participants were advised to weigh themselves in the morning with no clothing and before eating or drinking. The study staff had access to the participant Fitbit accounts during the study and accessed weight values via these accounts. Percentage weight change from baseline was calculated for each participant by subtracting the follow-up weight from the baseline weight and dividing by the baseline weight.

### Statistical Analysis

Retention, engagement, diet tracking, acceptability, and weight loss were summarized using descriptive statistics. For variables that were normally distributed, we described distributions using means and SDs. For variables that were not normally distributed, we described distributions using medians and IQRs. We compared engagement and diet tracking by treatment condition using *F* tests or the Mann-Whitney *U* test, as appropriate. This pilot study was not powered for weight loss; thus, statistical tests were not used to compare groups on weight loss [26]. A participant became pregnant during the intervention and was not included in the weight loss outcomes. Four participants (1 in closed enrollment and 3 in additional open enrollment) did not provide follow-up weight; thus, if weight was available within 1 week of the follow-up week, that weight was used (1 participant); otherwise, the baseline weight was used (3 participants). We conducted a conventional content analysis using a data-driven inductive framework [25] of focus group data on acceptability. HT and JD developed a codebook based on themes emerging from participant responses. JD and HT independently coded responses, and discussions were used to achieve consensus on coding discrepancies. The interrater reliability (IRR) and Cohen κ statistics were calculated [27]. We summarized the frequency of the themes.

Furthermore, we performed two exploratory analyses: (1) we summarized the total volume of engagement in each condition in each week and compared the conditions using Mann-Whitney *U* tests and (2) we tested whether the total volume of engagement in the Facebook group each week was associated with weekly weight loss among the randomized participants. To this end, we first used multiple imputation [33] to impute the missing values of weekly weight during the intervention for all randomized participants (excluding the participant who became pregnant). A total of 12.97% (164/1264) observations were missing and thus imputed. We then used the percentage weight change for each participant in each week as the outcome and the total volume of engagement (ie, number of posts, comments, and poll votes from participants and counselors) in the Facebook group in each week as the key predictor of interest and controlled for the treatment condition; week of the intervention; participants’ individual engagement in each week (log-transformed); baseline weight; and sociodemographic characteristics such as age, race, gender, and employment status in a linear mixed-effects model [38].



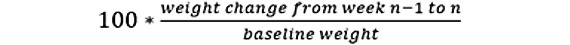



Data management and quantitative analyses were conducted using SPSS Statistics (version 26; IBM Corp). STATA (version 16.0; StataCorp LLC) was used for exploratory analysis of engagement volume and weight loss.

### Ethical Considerations

This research was approved by the University of Connecticut Institutional Review Board (H17-215) in October 2017.

## Results

### Overview

**Figure 1:**
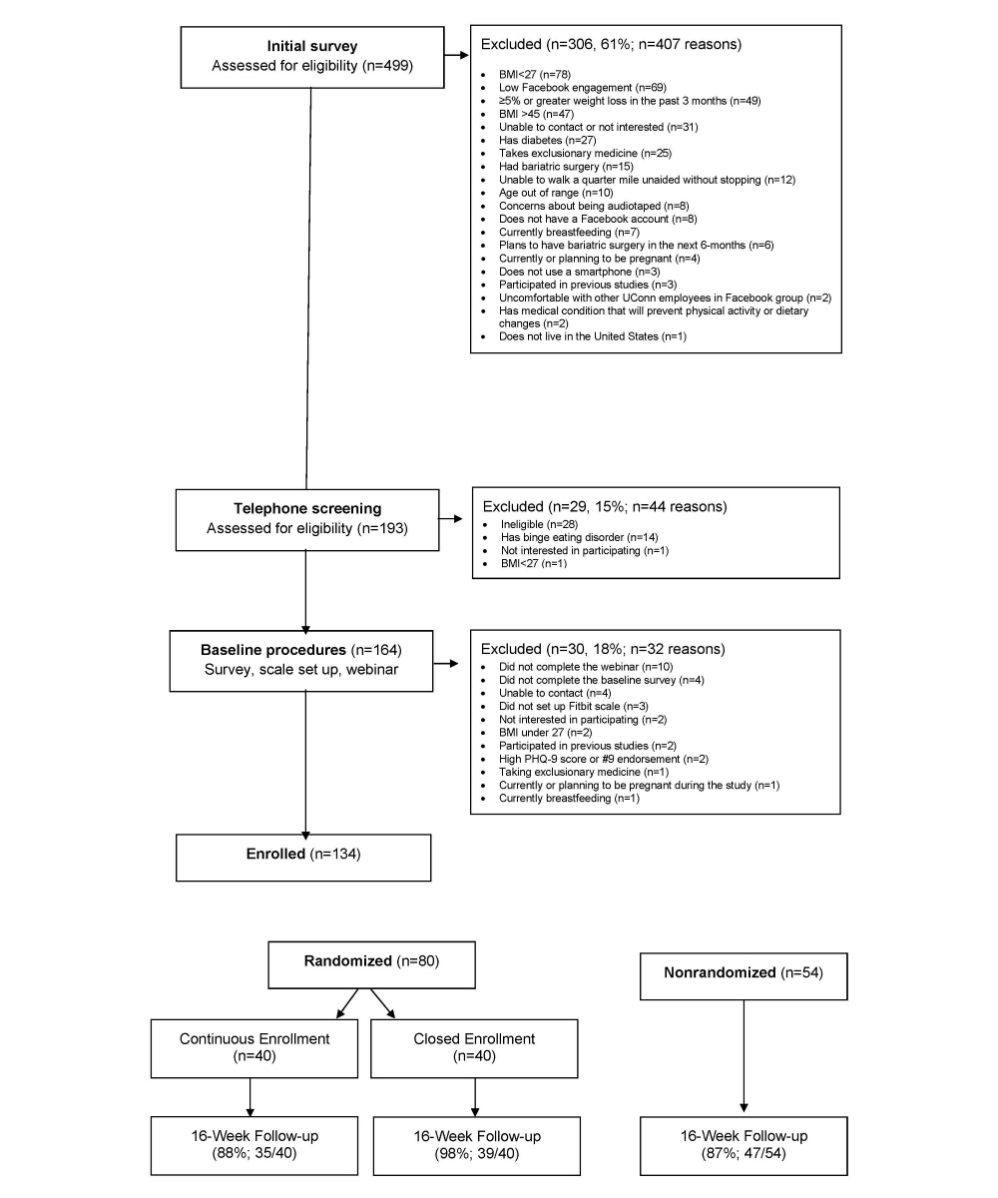
CONSORT (Consolidated Standards of Reporting Trials) diagram.

In total, 499 individuals completed the initial screening survey (Figure 1). Among those screened for eligibility, the most common reasons for exclusion were BMI out of range (<27 or >45 kg/m^2^), nonregular Facebook use, >5% weight loss in the past 3 months, and unresponsiveness to contact (Figure 1). A total of 80 participants were randomized to the 2 treatment conditions (Table 1). Overall, randomized participants were on average, aged 40.2 (SD 11.2) years with a baseline BMI of 34.4 (SD 5.0) kg/m^2^, 85% (68/80) were women, and 90% (72/80) were non-Hispanic White. The participants were from 34 US states and District of Columbia. A total of 54 participants were enrolled in the open enrollment condition between weeks 1 and 8. Of these 54 participants, a total of 19 (35%) joined during week 1, 7 (13%) joined during week 2, 3 (6%) joined during week 3, 8 (15%) joined during week 4, 7 (13%) joined during week 5, 5 (9%) joined during week 6, 3 (6%) joined during week 7, and 2 (4%) joined during week 8.

**Table 1:** Characteristics of enrolled participants, overall and by treatment condition.

		Closed enrollment (n=40)	Open enrollment; randomized (n=40)	Open enrollment; additional (n=54)	Overall (n=134)
Age (years), mean (SD)		40.4 (11.8)	40.0 (10.6)	40.3 (10.6)	40.2 (11.0)
Sex (female), n (%)		34 (85)	34 (85)	50 (93)	118 (88)
Baseline BMI (kg/m^2^), mean (SD)		34.8 (5.4)	34.0 (4.6)	34.5 (4.3)	34.4 (4.7)
Ethnicity (Hispanic or Latino), n (%)		3 (7.7)	1 (2.5)	4 (7.4)	8 (6)
**Race, n (%)**					
	White	36 (90)	36 (90)	44 (81.5)	116 (86.6)
	Black or African American	3 (7.5)	3 (7.5)	4 (7.4)	10 (7.5)
	Asian	0 (0)	0 (0)	2 (3.7)	2 (1.5)
	Native Hawaiian or other Pacific Islander	0 (0)	0 (0)	0 (0)	0 (0)
	American Indian or Alaska Native	0 (0)	0 (0)	0 (0)	0 (0)
	Multiethnic	0 (0)	1 (2.5)	3 (5.6)	4 (3)
	Unknown	1 (2.5)	0 (0)	1 (1.9)	2 (1.5)
**Marital status, n (%)**					
	Married or living with partner but not married	29 (72.5)	30 (75)	37 (68.5)	96 (71.6)
	Single	8 (20)	6 (15)	9 (16.7)	23 (17.2)
	Widowed, divorced, or separated	3 (7.5)	4 (10)	8 (14.8)	15 (11.2)
**Education, n (%)**					
	Less than high school or high school degree or equivalent	1 (2.5)	2 (5)	3 (5.6)	6 (4.5)
	Trade or technical or some college or Associate’s degree	8 (20)	11 (27.5)	14 (25.9)	33 (24.6)
	Bachelor’s degree or some graduate school	21 (52.5)	17 (42.5)	22 (40.7)	60 (44.8)
	Graduate degree	10 (25)	10 (25)	15 (27.8)	35 (26.1)
**Employment status, n (%)**					
	Employed full-time	28 (70)	27 (67.5)	35 (64.8)	90 (67.2)
	Employed part-time	7 (17.5)	4 (10)	10 (18.5)	21 (15.7)
	Student	2 (5.1)	2 (5)	2 (3.7)	6 (4.5)
	Unemployed or retired or disabled or homemaker	3 (7.5)	6 (15)	7 (13)	16 (12)
Posts or comments on Facebook daily, n (%)		20 (50)	18 (45)	25 (46)	63 (47)

### Retention

Retention exceeded the 80% benchmark in both treatment conditions, with 88% (35/40) of randomized open enrollment participants, 98% (39/40) of closed enrollment participants, and 87% (47/54) of additional open enrollment participants, providing complete follow-up data.

### Acceptability

Among participants who completed the follow-up survey (121/134, 90.3%), general acceptability ratings exceeded the 80% benchmark in both conditions, such that 92% (36/39) of the closed enrollment participants said they were a little to very likely to recommend the program to a friend, compared with 89% (31/35) of randomized open enrollment participants and 94% (44/47) of the additional open enrollment participants. When asked about the volume of posts by other participants in the group, the benchmark of at least 80% of the participants felt that other participants did not post too much was exceeded, such that 92% (36/39) of the closed enrollment participants said they either liked the amount of posts (30/39, 77%) or wanted more posts (6/39, 15%) by other participants compared with 86% (30/35) of the randomized open enrollment participants who said they either liked the amount of posts (22/35, 63%) or wanted more (8/35, 23%) and 92% (43/47) of the additional open enrollment participants who said they either liked (33/47, 70%) or wanted more (10/47, 21%; Table 2).

**Table 2:** Acceptability of post and reply volume by other participants.

		Closed enrollment (n=39), n (%)	Open enrollment, n (%)				All participants (n=121), n (%)
			Randomized (n=35)	Additional (n=47)	All participants (n=82)		
**Participant post volume**							
	I would prefer that participants did not post at all.	0 (0)	1 (2.9)	2 (4.3)	3 (3.7)	3 (2.5)	
	I would prefer fewer posts by participants.	3 (7.7)	4 (11.4)	2 (4.3)	6 (7.3)	9 (7.4)	
	I liked the amount of posts by participant.	30 (76.9)	22 (62.9)	33 (70.2)	55 (67.1)	85 (70.2)	
	I would prefer more posts by participants.	6 (15.4)	8 (22.9)	10 (21.3)	18 (22)	24 (19.8)	
**Participant reply volume**							
	I would prefer that participants did not comment/reply to posts at all.	0 (0)	2 (5.7)	1 (2.1)	3 (3.7)	3 (2.5)	
	I would prefer fewer comments/replies by participants.	2 (5.1)	3 (8.6)	1 (2.1)	4 (4.9)	6 (5)	
	I liked the amount of comments/replies by participants.	27 (69.2)	25 (71.4)	37 (78.7)	62 (75.6)	89 (73.6)	
	I would prefer more comments/replies by participants.	10 (25.6)	5 (14.3)	8 (17)	13 (15.9)	23 (19)	

When asked about the volume of replies by other participants in the group, the 80% benchmark (ie, 80% not feeling like other participants replied too much) was exceeded such that 95% (37/39) of the closed enrollment participants said they liked the amount of posts (27/39, 70%) or wanted more (10/39, 26%), whereas 85% (30/35) of the randomized open enrollment participants said they either liked the amount of posts (25/35, 71%) or wanted more (5/35, 14%), and 96% (45/47) of the additional open enrollment participants said they either liked the amount of posts (37/47, 79%) or wanted more (8/47, 17%; Table 2).

When asked to rate the responsiveness of counselors to participants’ posts, the 80% benchmark was not met in all groups: although 87% (34/39) of the closed enrollment participants said they were very responsive, only 77% (27/35) of the randomized open enrollment participants and 83% (39/47) of the additional open enrollment participants did so. Finally, when asked if they became Facebook friends with fellow participants, only 3% (1/39) of randomized participants in the closed enrollment and 3% (1/35) of open enrollment conditions had done so, whereas 15% (7/47) of the additional participants in the open enrollment condition had done so. The closed enrollment randomized participant who said yes to this question made 1 new Facebook friend, whereas the open enrollment participant had made 3 new Facebook friends, and of the 7 additional open enrollment participants who made new Facebook friends, 5 (71%) said they made 1 new Facebook friend, and 2 (29%) said they made 2 new Facebook friends.

Participants (118/134, 88.1%) who attended postintervention focus groups provided 165 responses to the question about what they liked most about the program (IRR=90%; Cohen κ=0.88; Table 3). The closed enrollment participants (n=37) provided 54 responses, the most common themes of which were sense of community (17/54, 32% responses), program content (16/54, 30% responses), and accountability (9/54, 17% responses). The open enrollment of randomized participants (n=34) provided 42 responses. The most common themes were sense of community (12/42, 29%), followed by accountability (10/42, 24% responses), and program content (10/42, 24% responses). The additional open enrollment participants (n=47) provided 69 responses. The most common themes were sense of community (22/69, 32% responses), accountability (17/69, 25% responses), and program content (15/69, 22% responses). Participants provided 123 responses regarding what they liked the least about the program (IRR=91.1%; Cohen *κ*=0.89). The closed enrollment participants (n=37) provided 40 responses, the most common themes of which were guidance that was not individualized enough (9/40, 23% responses), calorie tracking (8/40, 20% responses), and difficulty keeping up with the pace of the program (7/40, 18% responses). Randomized open enrollment participants (n=34) provided 37 responses. The top 3 most common themes of responses were difficulty feeling connected to the group (12/37, 32% responses), guidance not individualized enough (11/37, 30% responses), and problems with the technology (5/37, 14% responses). The additional open enrollment participants (n=47) provided 46 responses, the most common themes of which were guidance that was not individualized enough (9/46, 20% responses), calorie tracking (9/46, 20% responses), and difficulty keeping up with the pace of the program (8/46, 17% responses).

**Table 3:** Postintervention focus group data on intervention acceptability.

			Closed enrollment (n=37), n (%) responses		Open enrollment, n (%) responses					All participants (n=118), n (%) responses
					Randomized (n=34)		Additional (n=47)			
**Liked best**			54 (100)		42 (100)		69 (100)		165 (100)	
	Sense of community	17 (31.5)		12 (28.6)		22 (31.9)		51 (30.9)		
	Accountability	9 (16.7)		10 (23.8)		17 (24.6)		36 (21.8)		
	Program content	16 (29.6)		10 (23.8)		15 (21.7)		41 (24.8)		
	Convenience	6 (11.1)		5 (11.9)		5 (7.2)		16 (9.6)		
	Counselor feedback	6 (11.1)		4 (9.5)		9 (13)		19 (11.5)		
	Other	0 (0)		1 (2.4)		1 (1.4)		2 (1.2)		
**Liked least**			40 (100)		37 (100)		46 (100)		123 (100)	
	Difficulty feeling connected to the group	2 (5)		12 (32.4)		7 (15.2)		21 (17)		
	Not individualized enough	9 (22.5)		11 (29.7)		9 (19.6)		29 (23.6)		
	Technology problems	6 (15)		5 (13.5)		4 (8.7)		15 (12.2)		
	Calorie tracking	8 (20)		4 (10.8)		9 (19.6)		21 (17.1)		
	Pace was too fast	7 (17.5)		2 (5.4)		8 (17.4)		17 (13.8)		
	Weekly weigh-ins	2 (5)		2 (5.4)		3 (6.5)		7 (5.7)		
	Need more accountability	2 (5)		1 (2.7)		3 (6.5)		6 (4.9)		
	Nothing	4 (10)		0 (0)		3 (6.5)		7 (5.7)		

### Engagement

Among the randomized open enrollment participants, the median total engagement (reactions, replies or comments, and poll responses) over 16 weeks per participant was 77 (IQR 29.5-271.5), which was not statistically significantly different from 116.5 (IQR 29-173; *U*=763; *P*=.72) in the closed enrollment condition (Table 4). Because the additional open enrollment participants were in the group for 8-15 weeks, we could not compare their engagement data to the other groups. As expected, given the difference in the size of the 2 groups, the total volume of engagement from participants per week was higher in the open enrollment condition (n=94; median 229, IQR 129.5-336.5) than in the closed enrollment condition (n=40; median 125.5, IQR 86.5-188.5; *U*=64; *P*=.02). The total volume of engagement from both participants and counselors per week was also higher in the open enrollment condition (median 385, IQR 228-536.5) than in the closed enrollment condition (median 215, IQR 145.5-292; *U*=56; *P*=.007).

In terms of engagement in the year following the intervention, the open enrollment condition, including both randomized and additional participants (n=94), produced 4.78 times greater total engagement (sum=1266) than the closed enrollment group (n=40; sum=265). In the open enrollment condition, 43% (40/94) of the participants engaged at least once in the subsequent year, compared with 60% (24/40) of participants in the closed enrollment condition (N=134; *χ^2^*_1_=3.4; *P*=.06). A comparison of all 3 sets of participants (closed enrollment, randomized open enrollment, and additional open enrollment) on the proportion of participants who participated in the year following the intervention revealed no differences (24/40, 60%; 17/40, 43%; 23/54, 43%), respectively; N=134; *χ*^2^_2_=3.4; *P*=.18).

**Table 4:** Median total engagement per randomized participant during the 16-week intervention.

	Closed enrollment, (n=40), median (IQR)	Open enrollment randomized, (n=40), median (IQR)	Mann-Whitney *U* test	*P* value
Posts	1.5 (0-5)	1 (0-3.5)	758.5	.68
Replies	39 (9.5-73.5)	36.5 (14.5-80.5)	778.0	.83
Reactions	28 (7.5-81.5)	29 (7-134.5)	727.5	.49
Poll votes	12 (5.5-20.5)	10.5 (5-19.5)	763.0	.72
Total engagements	116.5 (28.5-174.0)	77 (29.5-271.5)	763.0	.72

### Diet Tracking

Randomized participants (n=40) in the open enrollment condition tracked their diet on a mean of 42.4 (SD 33.0) days out of the 84 days, and participants in the closed enrollment condition (n=40) tracked their diet on a mean of 36.3 (SD 34.7) days, which represented an average of 38% (SD 30%) of possible days for participants in the open enrollment condition and 32% (SD 31%) of possible days for participants in the closed enrollment condition (*F*_1,79_=0.653; *P*=.42). Because the additional open enrollment participants were in the group anywhere from 56 to 105 days, we could not compare their diet tracking data to the other groups.

### Counselor Engagement

In terms of counselor engagement, counselors produced 7653 total engagements in the open enrollment condition, which was about twice as many as the counselors in the closed enrollment condition, where counselors produced 3618 total engagements (reactions or likes, comments, and posts; Table 5). In terms of counselor reactions or likes, the open enrollment counselors produced 5018 which was 2.27 times higher than the closed enrollment condition counselors who produced 2203 during the intervention. In terms of counselor comments, open enrollment counselors produced 2392 which was approximately twice that of the closed enrollment condition counselors who produced 1153 comments during the intervention. In terms of counselor posts, 224 were prescheduled. In addition, the open enrollment counselors produced 19 other posts during the intervention, and the closed enrollment condition counselors produced 38. On average, each week counselors generated 478.31 (SD 284.57) total engagements in the open enrollment condition and 226.13 (SD 86.69) total engagements in the closed enrollment condition (*U*=38; *P*<.001).

**Table 5:** Total counselor engagement during the 16-week intervention.

	Closed enrollment, n (%)	Open enrollment, n (%)	Difference between open and closed enrollment (%)
Preprogramed intervention posts	224 (6.2)	224 (2.9)	0
Other posts	38 (1.1)	19 (0.2)	−100
Comments	1153 (31.9)	2392 (31.3)	+207.5
Reactions	2203 (60.9)	5018 (65.6)	+227.8
Total	3618 (100)	7653 (100)	+211.5

### Weight Loss

Over 16 weeks, participants randomized to the open enrollment condition (n=40) lost an average of −6.67 (SD 9.84) pounds or −3.08% (SD 4.28%) of their baseline weight and participants randomized to the closed enrollment condition (n=40) lost an average of −4.47 (SD 9.54) pounds or −1.87% (SD 4.41%) of their baseline weight. In terms of clinically significant weight loss (ie, ≥5% of baseline weight), 30% (12/40) and 18% (7/40) of participants randomized to the open enrollment and closed enrollment conditions, respectively, achieved ≥5% weight loss. The 54 additional participants in the open enrollment condition lost a mean of 2.8% (SD 4.5%) of their baseline weight and 20% (11/54) achieved ≥5% weight loss over a median of 13 (IQR 11-15) weeks they were in the group.

### Relationship Between Volume of Engagement and Weight Loss Among Randomized Participants

Participants’ individual engagement was the strongest predictor of weight loss; a 100% increase in individual engagement each week was associated with an additional 0.11% weekly weight loss (*P*<.001; 95% CI 0.05%-0.16%). However, the total volume of engagement was also associated with weight loss such that every 100 engagements in the Facebook group each week were associated with an additional 0.1% weekly weight loss for each randomized participant (*P*=.02; 95% CI 0.02%-0.18%), after controlling for individual engagement, treatment condition, time, baseline weight, and sociodemographic characteristics (eg, age, race, gender, and employment status).

## Discussion

### Principal Findings

The open enrollment approach to conducting a Facebook-delivered weight loss intervention allows members to flow into the group throughout the program, and has the potential benefits of treating more people at once and producing a higher volume of content overall. The possible trade-offs are that members might find the feed too busy or they may be dissatisfied with the amount of attention they receive from the counselor. We tested the feasibility of the open enrollment approach and discovered that it was feasible and acceptable relative to the typical approach to group-based lifestyle interventions, in which a specific number of participants are enrolled all at once and begin and end the program at the same time. We enrolled 54 additional participants in the open enrollment condition over the first 8 weeks of a 16-week intervention, bringing the total group size to 94. Despite the open enrollment group more than doubling in size during the study, the outcomes of retention and acceptability in both conditions exceeded the 80% benchmarks, with the exception that only 77% (27/35) of randomized open enrollment participants felt that the counselors were very responsive. However, 83% (39/47) of the additional open enrollment participants in that group felt that counselors were very responsive. It is possible that some randomized participants perceived a reduction in counselor responsiveness as the group size increased. Despite this, the diet tracking frequency among randomized participants was similar across both conditions, which means that the open enrollment approach did not appear to negatively impact adherence to this key behavioral strategy. Counselors in the open enrollment condition had just over twice the engagement as the closed enrollment condition, which is consistent with the finding of a greater volume of participant engagement in the open enrollment condition. Interestingly, participants who were enrolled in the open enrollment condition while it was ongoing lost similar amounts of weight as both the randomized participants in that condition and the closed enrollment condition (mean 2.8%, SD 4.5%; mean 3.1%, SD 4.3%; and mean 1.9%, SD 4.4%, respectively), even though they were in the group for a mean and median of approximately 13 (IQR 11-15) weeks. Because this study was not powered to detect group differences in weight loss, a fully powered trial is needed to determine if the open enrollment approach can produce greater weight loss outcomes.

Contrary to our hypothesis, the randomized open enrollment participants did not engage significantly more than the closed enrollment participants did. This means that the greater volume of content in that group did not prompt the original randomized participants to post or reply more often. As such, if open enrollment proved to be more efficacious than closed enrollment for weight loss in a fully powered randomized trial, it would seem unlikely that higher individual engagement would be the mechanism of action. Future studies should explore whether groups differ in terms of intervention content, which can be thought of as both a form of passive engagement and intervention receipt. Although we did not observe group differences in individual engagement, as in previous studies [21], participant engagement was a predictor of weight loss outcomes. The open enrollment condition as a whole had a significantly greater overall volume of engagement (from participants and counselors combined) than the closed enrollment condition, likely because of the larger size of the group. The total volume of engagement in the group each week was also a predictor of weight loss each week, suggesting that a busier group may benefit individual members of that group. Alternatively, this finding could reflect that participants who are more successful with their diet and exercise habits engage more often in those weeks. Regardless, a Facebook group with a higher volume of engagement will rank higher in Facebook’s newsfeed algorithm for any given group member, especially for group members who engage regularly [30]. This would result in greater intervention receipt, which could be a possible mechanism of action should a fully powered trial reveal the open enrollment approach to be advantageous for weight loss. Facebook’s newsfeed algorithm is also influenced by the extent to which participants engage with each other and even more so if that engagement is with other Facebook friends. A small number of group members (n=9) made new Facebook friends, while in the study, and most (8/9, 89%) were in the open enrollment condition. Future research is needed to determine how meaningful interactions among participants in Facebook-delivered interventions can be facilitated.

Although the open enrollment condition had a significantly greater volume of engagement in their group, 86% (30/35) of those randomized to this group and 91% (43/47) of the additional participants in this group said they either liked the amount of posts by other participants or wanted more, and 85% (30/35) of randomized and 96% (45/47) of additional participants in this group said they either liked the amount of comments by other participants or wanted more. This further supports the notion that a Facebook weight loss group size of 94 is feasible when ushering new participants gradually over time. Notably, 25% (10/39) of the participants in the closed enrollment condition said they would have preferred other participants to comment more, whereas only 13% (5/35) of the randomized and 17% (8/47) of the additional participants in the open enrollment condition said so. A randomized trial of a hybrid web-based weight loss program with monthly in-person groups compared group sizes of 20 and 100 and found no differences in weight loss among groups and high satisfaction in both conditions [39]. This suggests that groups as large as 100 participants do not seem to have deleterious effects on outcomes or feasibility when using web-based or hybrid approaches, regardless of whether participants start at the same time or are continuously enrolled. However, a trial comparing groups of 10 to 30 for an in-person weight loss program found that participants in the smaller groups lost significantly more weight than those in larger groups [40]. In the study, smaller groups had better session attendance, which was a significant predictor of weight loss outcomes. Group cohesion might be stronger in smaller in-person groups versus larger in-person groups because group meetings are the only opportunity to bond when the meetings are in person, and the more people who are in the room, the less time any one participant gets to talk. In web-based weight loss interventions, group cohesion may be less dependent on group size because opportunities for participant interaction are not limited to a single 90-minute weekly meeting; rather, opportunities are available 24/7.

In the postintervention focus groups, participants were asked what they liked the most about the program and in all 3 groups of participants (open enrollment randomized, open enrollment additional, and closed enrollment). The most common response was a sense of community, comprising 28.6% to 31.9% of responses in each group. This is further evidence that a web-based group of 94 people was not too large for participants to feel a sense of community. However, when asked what they liked the least about the program, 32% (12/37) of open enrollment randomized participant responses said they had difficulty feeling connected to the group compared with 5% (2/40) of the closed enrollment participant responses. This suggests that the entry of new participants into the open enrollment group may have disrupted the dynamics for some of the randomized participants; however, far fewer nonrandomized participant responses reflected this (7/46, 15%). Activities that facilitate group cohesion may be useful in larger groups, regardless of whether the participants start at the same time or are continuously enrolled. For example, small breakout sessions, icebreakers, or a buddy system may be used to help group members get to know each other better.

Future research is needed to determine the extent to which web-based weight loss groups can grow while still being feasible and acceptable to the participants. Although open enrollment did not result in the originally randomized participants engaging more than the closed enrollment group, the ability to treat many patients at once certainly has important advantages in terms of scalability; however, this should not be done at the expense of group cohesion and undue cognitive burden. The open enrollment approach may be more feasible in real-world settings for 2 reasons. First, multiple small groups may become more difficult to manage administratively than fewer large groups, and second, patients will not have to wait until enough people are enrolled to begin treatment. For example, in our study, randomized participants had to wait on average nearly 30 days from when they provided baseline data to start the intervention, because we needed to recruit, screen, and onboard 80 participants before we could randomize them into their groups. An open enrollment approach that allows people to start the program immediately might take better advantage of the heightened motivational state that prompted patients to enroll in the first place. It might also prevent any patient loss that may occur during the waiting period.

### Limitations

This study had some limitations. First, the sample was predominantly non-Hispanic White and female; thus, the results may not be generalizable to other groups. Historically, lifestyle interventions have been plagued by low enrollment of men [41]. Future research should explore men’s perspectives on participation in behavioral programs on Facebook. Second, weight loss was modest, and the study was not powered to detect group differences in weight loss. Weight loss was similar to other social media-delivered weight loss interventions in similar samples [15,42]. A fully powered trial is needed to establish the efficacy of this intervention approach in weight loss outcomes. Third, this study did not assess the time counselors spent delivering the intervention in each condition, which prevented us from calculating and comparing the cost of conducting each condition. However, we found that counselors in the open enrollment condition had twice as many comments as those in the closed enrollment condition, which indicates that they likely put more time into their group. Future trials should perform cost-effectiveness analyses to determine if any benefit of the open enrollment approach to a larger group of patients is worth the extra costs associated with the extra time spent by counselors. A previous study found that web-based weight loss intervention cost US $67.74 per kilogram lost compared with US $88.31 per kilogram lost in an in-person weight loss intervention, where groups were similarly sized (12-18 participants) [43]. This highlights the importance of further optimizing web-based approaches given that they are more cost-effective than traditional approaches. Even if counselors spent the same amount of time per participant in the larger versus smaller group, open enrollment may still be more cost-effective when treating the same number of people because of the extra time needed in the closed enrollment groups to create additional Facebook groups for each set of 40 people and scheduling posts in those groups. Finally, we did not assess whether the conditions differed in terms of the number of participants who muted notifications from the group, which is a potentially important outcome for studies testing group size. Future studies should assess this because participants might be more likely to mute notifications in a very busy group, and muting notifications could impact intervention receipt and outcomes.

### Conclusions

Lifestyle interventions are effective, but traditional delivery modalities (eg, in-person or web-based group meetings) suffer from poor scalability. Web-based approaches that can efficiently serve a large number of patients are needed. We found that the approach of continuously enrolling participants in an ongoing web-based program was feasible and acceptable. Future research should explore the cost-effectiveness of enrolling large numbers of patients in web-based programs using an open enrollment approach that eliminates waiting times and leverages strategies to facilitate group cohesion.

## Abbreviations

DPP: Diabetes Prevention Program
IRR: interrater reliability
